# Prevalence of metabolic syndrome in low-income childhood-onset systemic lupus erythematosus patients

**DOI:** 10.1186/s42358-025-00464-5

**Published:** 2025-07-07

**Authors:** Natalia Gomes Iannini, Carlos Ewerton Maia Rodrigues

**Affiliations:** 1 Graduate Program in Medical Sciences, University of Fortaleza (Unifor), Fortaleza, Brazil; 2 Department of Internal Medicine, Federal University of Ceará , Fortaleza, Ceará , Brazil; 3 Rheumatology Division, Hospital Geral de Fortaleza, Fortaleza, Brasil

**Keywords:** Pediatric systemic lupus erythematosus, Metabolic syndrome, Cardiovascular risk, Children

## Abstract

**Objective:**

To determine the prevalence of metabolic syndrome (MetS) in patients with childhood onset Systemic Lupus Erithematosus (cSLE) and controls from Northeastern Brazil and to verify its association with specific SLE parameters and cardiovascular risk factors.

**Methods:**

The prevalence of MetS was assessed cross-sectionally in 58 patients with cSLE and 33 age -matched controls. Information was collected by clinical examination and standardized questionnaires, investigating personal and family history of cardiovascular disease and obesity and socioeconomic and demographic characteristics.

**Results:**

The prevalence of MetS was higher in cSLE patients than in controls according to both ABRAN criteria (8.6% vs. 0%; *p* = 0.083) and IDF criteria (10,3% vs. 3.0%; *p* = 0.208), but without statistical significance. Importantly, 91.4% of patients were from a low-income household. Patients with MetS according to ABRAN also had lower ESR levels (*p* = 0.039), higher total cholesterol (*p* = 0.013), HDL-c (*p* = 0.007) and triglycerides (*p* = 0.001) and a lower albumin level (*p* = 0.016). Patients with MetS according to IDF had higher SDI scores (*p* = 0.039) and higher C3 and C4 levels (*p* < 0.001 and *p* < 0.001, respectively). The multivariate logistic regression identified higher levels C4 (OR = 32.6; 95% CI = 1.0-544.0; *p* = 0.015) and increase in the number of leukocytes (OR = 1.9, 95%CI = 1.1–3.2; *p* = 0.022) as independent risk factors for MetS in patients with cSLE.

**Conclusion:**

The prevalence of Mets in the patients with cSLE seems to be low in this population. There was association of MetS with higher cumulative damage indices and levels of complement. We did not observe any association with clinical manifestations, autoantibody profile and dose of corticosteroids.

## Background

Systemic Lupus Erythematosus (SLE) is a prototypic systemic autoimmune disease, characterized by the presence of multiple autoantibodies, that can affect any organ or system [1]. Ten to 20% of patients begin symptom presentation before the age of 18, in which cases the term ‘childhood onset SLE’ (cSLE) is applied [1, 2].

Therapeutic advancements for cSLE have significantly increased survival rates, with patients often reaching adulthood. However, these individuals may also accumulate comorbidities, either due to damage from the disease itself or adverse effects from treatment, namely corticosteroids. The primary causes of death are still acute complications of the disease, such as infection, kidney failure, or cardiopulmonary disease. Conversely, in adult life, the risks shift towards cardiovascular disease and cancer, posing major concerns [1, 2].

Metabolic syndrome (MetS) is defined by the presence of biochemical, clinical, and metabolic abnormalities that predispose individuals to the development of type-2 diabetes mellitus and an increased cardiovascular risk. The triggering event is insulin resistance (IR), and its main risk factors include overweightness and obesity [3, 4]. The diagnosis of MetS can be established through diagnostic criteria, but there is currently no consensus on which criterion is more suitable for use in children and adolescents [3]. In an effort to standardize the classification of MetS in Brazilian children and adolescents, the Brazilian Association of Nutrology (ABRAN) published a consensus in 2020, introducing new criteria for diagnosing MetS [5].

A systematic review with meta-analysis, including 47 studies, concluded that the prevalence of MetS in patients with SLE was 26% and that these patients have a greater predisposition to develop MetS compared to the control population. Notably, the analysis included only one study carried out with the pediatric population [6]. It involved 37 patients with cSLE, revealing a prevalence of 32% in patients with cSLE as opposed to 11% in control patients [7]. Another study, which assessed the prevalence of MetS in 76 southeastern Brazilian adolescents with SLE, identified a higher prevalence of MetS in this population (18.4% by NCEP-ATP III, 17.1% by IDF, and 2.6% by WHO) than in the controls (none of the control patients met the criteria for MetS) [8]. To the best of our knowledge, these are the only studies investigating MetS in patients with cSLE.

The aim of this study was to ascertain the prevalence of MetS among cSLE patients in a tertiary healthcare center in northeastern Brazil compared with age-matched healthy controls and evaluate the possible relationship between MetS and sociodemographic, clinical, and laboratory variables and cardiovascular risk predictors in cSLE patients.

## Methods

### Patients and controls

The present cross-sectional study involved patients with cSLE that were consecutively recruited during their routine consultations at the Pediatric Rheumatology outpatient clinic of a tertiary hospital in northeastern Brazil between December 2022 and May 2023. The inclusion criteria comprised patients aged ≥ 10 years old who met the classification criteria for SLE according to ACR 1997 [9], with disease onset before the age of 18. Individuals with overlapping systemic autoimmune disease (i.e., Sjögren’s disease, antiphospholipid antibody syndrome) were excluded. Healthy volunteers were recruited from local community and controls that had any history of chronic, cardiovascular, and autoimmune disease were excluded. This research project received approval from the hospital’s ethics committee via *Plataforma Brasil* (CAAE No.: 66384222.8.0000.5042). Written consent forms were signed by all participants and their guardians. The study was conducted in accordance with the guidelines of the Declaration of Helsinki.

### Data collection

At the time of recruitment, data were collected through clinical examination and standardized questionnaires. Information regarding the presence of diabetes mellitus (DM), systemic arterial hypertension (SAH), lifestyle, personal and family history of cardiovascular disease and obesity, the time since symptom onset and diagnosis, and sociodemographic characteristics was also recorded.

Individuals engaged in less than 150 min of moderate-intensity exercise per week were considered sedentary [10]. DM was defined according to the Brazilian Society of Diabetes, wherein the diagnosis was confirmed by two abnormal tests showing fasting plasma glucose ≥ 126 mg/dL, blood glucose ≥ 200 mg/dL two hours after a 75 g anhydrous glucose overload, or the presence of unequivocal hyperglycemia symptoms, or HbA1c ≥ 6.5% [11]. SAH was considered present when blood pressure was persistently above the 95th percentile for age, height, and sex in patients younger than 13 years of age, systolic blood pressure (SBP) > 130 mmHg, and/or diastolic blood pressure (DBP) > 80 mmHg in patients older than 13 years of age [10]. Alternatively, the use of antihypertensive drugs at the time of the evaluation also indicated SAH. Overweightness and obesity were classified according to the WHO definitions for those aged 5 to 19 years old, where overweightness is considered when the body mass index (BMI) is between the z-score + 1 and + 2, obesity between + 2 and + 3, and severe obesity when above the z-score + 3 [4].

Anthropometric measurements were taken at the beginning of the study. A standard digital scale was used for weight, and a wall-mounted stadiometer was used to assess height. These measurements were used to calculate the BMI. The waist circumference (WC) was measured at midpoint between the tenth rib and the superior iliac crest [4], while blood pressure was measured at rest using a sphygmomanometer appropriate for the size and age of each individual.

For the laboratory analysis, blood samples were collected after 8–12 h of fasting, and midstream first morning spot urine was used.

Disease activity was assessed using the Systemic Lupus Erythematosus Activity Index 2000 (SLEDAI-2 K) [12], and the Slicc damage index score (SDI) [13] was adopted to estimate damage. The cumulative dose of corticosteroids used was obtained by the careful review of medical charts and calculated by the sum of the daily dosages vs. the time (in days) of treatment. Doses of oral and parenteral corticosteroids were converted into equivalent doses of prednisone. Additionally, the current or previous use of immunosuppressants was recorded.

### MetS definition

Two sets of criteria were used to define MetS: IDF [14] and ABRAN criteria [5].

The definition of MetS according to IDF criteria comprised children aged 10 to < 16 years with a waist circumference (WC) ≥ 90th percentile or an adult cut-off if lower, and at least two of the following alterations: triglyceride (TG) levels ≥ 150 mg/dL, high density lipoprotein cholesterol (HDL-c) < 40 mg/dL, arterial BP ≥ 130/85 mmHg, and fasting plasma glucose ≥ 100 mg/dL. For children older than 16 years of age, the adult IDF criteria were adopted, with waist circumference ≥ 90 cm for men and ≥ 80 cm for women, along with at least two of the following criteria: TG levels ≥ 150 mg/dL or treatment for this abnormality, HDL-c < 50 mg/dL for women or < 40 mg/dL for men or treatment for this abnormality, arterial BP ≥ 130/85 mmHg or on antihypertensive medication, and fasting plasma glucose ≥ 100 mg/dL or previously diagnosed type-2 diabetes [14].

As for the definition of MetS according to ABRAN criteria, four domains were evaluated: adiposity (WC), glycemic profile (fasting glucose and insulin), lipid profile (HDL-c and triglycerides), and blood pressure (BP). Cut-off values were stratified based on ideal levels for age and sex [5]. The presence of alterations in three out of the four domains was classified as MetS.

### Low-income definition

For income classification, low-income families were those with an income of up to 3 Brazilian minimum wages or a *per capita* income of up to half a Brazilian minimum wage, as defined by the Brazilian Federal Government [15].

### Statistical analysis

Categorical data were presented as absolute counts and percentages, and associations between them were assessed using the chi-square test or Fisher’s exact test. Continuous data were initially analyzed for normality using the Shapiro-Wilk test, followed by histogram evaluation, Q-Q plots, and dispersion measures. Parametric data were expressed as mean ± standard deviation, and non-parametric data as median and interquartile range. Comparisons between two independent groups were conducted using Student’s t-test for parametric data and the Mann-Whitney test for non-parametric data.

To identify independent predictors of MetS in cSLE, multivariable logistic regression analysis was performed using two different definitions for the outcome: MetS according to IDF and according to ABRAN. For model building, bivariate analyses were conducted between each explanatory variable and the presence of MetS defined by IDF criteria. Variables with a p-value < 0.20 and more than 80% of valid data were considered eligible for multivariable analysis.

To avoid collinearity and overfitting, variables that are part of the diagnostic criteria for Mets, such as triglycerides, glycemia, insulin, and HDL-cholesterol were excluded. In addition to the selected candidate variables, age and sex were included based on their biological relevance. The final model included laboratory markers unrelated to the diagnostic criteria (e.g., inflammatory biomarkers) and clinical indicators of disease activity or classification, aiming to explore potential associations beyond the MetS components themselves. The analyses were performed using SPSS software for Macintosh (version 23.0. Armonk, NY, USA: IBM Corp.) and the R software, with p-values < 0.05 considered statistically significant.

## Results

Sixty-five patients with cSLE were initially recruited to participate in this study. However, five were excluded on account of overlapping diagnosis of other autoimmune diseases, such as antiphospholipid antibody syndrome and Sjögren’s disease, and two due to a lack of laboratory test results. In the end, 58 patients were evaluated, 87.9% of whom were female and 82.8% non-Caucasian, with a mean age of 16 years (standard deviation (SD) ± 2.6). In addition, the majority were considered of low income, with 91.4% having a family income of up to three minimum wages. The control group included 33 individuals, 72.7% of whom were female and 72.7% non-Caucasian, with a mean age of 14.9 ± 2.6 years (Table 1).

**Table 1:** Sociodemographic and metabolic characteristics of cSLE patients *versus* controls

	Control (*n* = 33)	cSLE patients (*n* = 58)	*p*-value*
**Sex**			0.067
Female, n (%)	24 (72.7)	51 (87.9)	
Male, n (%)	9 (27.3)	7 (12.1)	
**Age (years)**	14.9 ± 2.6	16.0 ± 2.6	0.062
**Ethnicity**			0.113
Non-Caucasian, n (%)	24 (72.7)	50 (86.2)	
Caucasian, n (%)	9 (27.3)	8 (13.8)	
**Household income**			< 0.001
Low income, n (%)	17 (51.5)	53 (91.4)	
**Lifestyle Habits**			
Physical activity, n (%)	12 (36.4)	18 (31.0)	0.603
**Family history**			
Cardiovascular Disease, n(%)	11 (33.3)	20 (34.5)	0.911
Obesity , n (%)	12 (36.4)	30 (51.7)	0.158
**Overweightness**, n (%)	6 (18.2)	14 (24.1)	0.804
**Obesity**, n (%) **MetS (IDF)**, n (%) **MetS (ABRAN)**, n (%)	5 (15.2) 1 (3.0) 0 (0)	8 (13.8) 6 (10.3) 5 (8.6)	0.804 0.208 0.083
**Waist Circumference (cm)**	74.9 ± 9.7	79.6 ± 12.5	0.046
**Glycemia (mg/dL)**	89.7 ± 8.3	84.0 ± 10,3	0.005
**Glycated hemoglobin**, n (%)	5.3 ± 0.4	5.4 ± 0.5	0.306
**Insulin**	8.9 (6.9; 11.1)	8.9 (5.5; 13.6)	0.909
**Total cholesterol (mg/dL)**	153.9 ± 32.9	158.1 ± 46.3	0.621
**HDL-c (mg/dL)**	51.5 ± 13.5	49.8 ± 16.3	0.592
**LDL-c (mg/dL)**	86.0 ± 27.4	87.6 ± 32.1	0.798
**Triglycerides (mg/dL)**	78.5 ± 32.0	104.2 ± 52.5	0.005

c-SLE: childhood onset Systemic Lupus Erithematosus; MetS: metabolic syndrome; IDF: International Diabetes Federation; ABRAN: Brazilian Association of Nutrology; HDL-c: high-density lipoprotein cholesterol; LDL-c: low-density lipoprotein cholesterol

Quantitative data expressed as mean ± standard deviation or as median and interquartile range in parentheses

* The Chi-square test or Fisher’s exact test was used for categorical data, and the Student’s t-test or Mann-Whitney test for continuous data

Regarding lifestyle, only 31% practiced physical activity, while alcohol consumption and smoking were not reported on the patients group.

A family history of cardiovascular disease and obesity was present in 34.5% and 48.3% of the sample, respectively.

In terms of clinical manifestations (Table 2), arthritis (77.6%) and hematological manifestations (67.2%) were most frequent. It was observed that 55.2% of the patients had lupus nephritis, 80% of whom underwent biopsy, and, among these, 60.7% were classified as class IV. Anti-DNA was the most documented antibody and was present in 72.4% of patients. The median time since diagnosis at the time of the evaluation was 33 months, and the median time since symptom onset was 40 months.

**Table 2:** Clinical characteristics of patients with cSLE

	*N* = 58
**Time to diagnosis (months)**	33.5 (13.3; 51.0)
**Duration of symptoms (months)**	40.0 (19.3; 60.5)
**Clinical Manifestations**, **n (%)**	
Skin rash ,	28 (48.3)
Discoid lupus erythematosus	4 (6.9)
Mucous ulcers	20 (34.5)
Arthritis	45 (77.6)
Serositis	23 (39.7)
Nephritis	32 (55.2)
Neurological	11 (19.0)
Hematological	39 (67.2)
**Antibodies**, **n (%)**	
Anti-Sm	24 (41.8)
Anti-DNA	42 (72.4)
Anti-Ro	22 (32.1)
Anti-La	7 (5.6)
Anti-RNP	25 (36.5)
Anti-P	6 (10.0)
B2GP1 IgM	13 (13.5)
B2GP1 IgG	9 (5.8)
ACL IgM	11 (19.0)
ACL IgG	7 (11.8)
Direct Coombs	28 (48.3)
LAC	18 (31.0)
**SLEDAI – 2 K**	4.0 (2.0; 8.0)
**SDI**	0.0 (0.0; 0.0)

c-SLE: childhood onset Systemic Lupus Erithematosus; B2GP1: anti-β-2-glycoprotein-1; ACL: anticardiolipin; LAC: lupus anticoagulant; SLEDAI: Systemic Lupus Erythematosus Disease Activity Index; SDI: Slicc damage index

Categorical data expressed as absolute counts and percentages in parentheses. Quantitative data expressed as mean ± standard deviation or as median and interquartile range in parentheses

*The chi-square test or Fisher’s exact test was used for categorical data and Student’s t-test for continuous data

All patients were using hydroxychloroquine, approximately 52% had used cyclophosphamide, 60% azathioprine, and other medications, including mycophenolate mofetil, cyclosporine, and methotrexate, were used less frequently. Six patients (10.3%) used rituximab, and one patient (1.7%) used belimumab. Almost all subjects (96.5%) had history of corticosteroids use and 48.3% were using at the time of evaluation. The median cumulative dose for corticosteroids was of 15,366.3 mg [ 9,358.2–25,599.4]. Overall, patients evaluated presented low level of disease activity and damage, represented by SLEDAI-2 K 4.0 [2.0–8.0] and SDI 0.4 ± 0.8, respectively.

The prevalence of MetS was higher in cSLE patients than in controls according to both ABRAN criteria (8.6% vs. 0%; *p* = 0.083) and IDF criteria (10,3% vs. 3.0%; *p* = 0.208), respectively, but without statistical significance. Only two cSLE subjects met simultaneously both MetS criteria. Significantly, higher levels of triglycerides were observed in cSLE patients compared to controls (78.5 mg/dL ± 32.0 vs. 104.2 mg/dL ± 52.5; *p* = 0.005). The detailed comparison of sociodemographic and metabolic characteristics of patients and controls is described in Table 1.

Characteristics of cSLE patients according to IDF and ABRAN MetS criteria are detailed in Tables 3 and 4, respectively. No differences were observed in sociodemographic data, or clinical manifestations between the groups with and without MetS, regardless of the adopted criteria.

**Table 3:** Characteristics of cSLE patients according to the presence of metabolic syndrome defined by IDF criteria

	Metabolic Syndrome (IDF)			
	**No** (*N* = 52^1^)	**Yes** (*N* = 6^1^)	p-value*	
**Sex**			0.555	
Female, n (%)	46 (88.5)	5 (83.3)		
Male, n (%)	6 (11.5)	1 (16.7)		
**Age (years)**	15.9 ± 2.6	16.7 ± 2.3	0.618	
**Ethnicity**			0.139	
Non-Caucasian, n (%)	44 (84.6)	4 (66.6)		
Caucasian, n (%)	8 (15.4)	2 (33.3)		
**Household income**			0.433	
Low income, n (%)	48 (92.3)	5 (83.3)		
**SBP**	109.2 ± 10.7	115.5 ± 15.3	0.283	
**DBP**	71.0 ± 9.7	73.5 ± 16.4	0.406	
**BMI (kg/m2)**	22.7 ± 4.7	27.1 ± 6.3	0.089	
**WC (cm)**	78.3 ± 12.2	91.1 ± 10.1	0.019	
**Overweightness**, **n (%)**	10 (19.2)	2 (33.3)	0.232	
**Obesity**, **n (%)**	3 (5.7)	2 (33.3)	0.232	
**Sedentary lifestyle**, ** n(%)**	35 (67.3%)	5 (83.3%)	0.655	
**Diagnosis time (months)**	43.8 ± 35.8	43.8 ± 35.8	0.118	
**Duration of symptoms (months)**	24.0 ± 19.0	24.0 ± 19.0	0.119	
**Current steroid dose (mg)**	0.0 (0.0; 20.0)	7.5 (0.0; 26.3)	0.620	
**Cummulative steroid dose (mg)**	15,366.3(10,576.3; 26,210.6)	12,872.5 (6,556.3; 23,235.0)	0.435	
**SLEDAI – 2 K**	5.9 ± 6.0	1,0 (0; 6.5)	0.290	
**SDI**	0.0 (0.0; 0.0)	1.0 (0.0; 2.8)	0.039	
**ESR (mm)**	24.7 ± 23.7	16.0 ± 12.1	0.515	
**CRP (mg/dL)**	0.9 (0.5; 1.7)	1.4 (0.8; 4.6)	0.721	
**Creatinine (mg/dL)**	0.8 ± 1.3	0.9 ± 0.4	0.075	
**Urea (mg/dL)**	22.5 (18.0; 34.3)	31.0 (21.8; 41.8)	0.307	
**Spot urine protein/creatinine ratio**	0.1 (0.1; 0.2)	0.1 (0.1; 1.4)	0.835	
**Fasting blood glucose (mg/dL)**	83.6 ± 10.6	87.3 ± 7.6	0.234	
**Glycated hemoglobin (%)**	5.4 ± 0.5	5.3 ± 0.2	0.398	
**Insulin (microIU/mL)**	9.3 (6.8; 13.7)	15.8 (10.9; 19.1)	0.051	
**Total cholesterol (mg/dL)**	154.0 ± 43.7	193.5 ± 57.5	0.105	
**HDL-c (mg/dL)**	49.1 ± 16.1	55.7 ± 18.3	0.414	
**LDL-c (mg/dL)**	85.8 ± 31.8	102.9 ± 33.0	0.206	
**Triglycerides (mg/dL)**	96.1 ± 39.6	174.7 ± 93.5	0.052	
**Albumin (g/dL)**	4.3 ± 0.4	4.1 ± 0.7	0.686	
**Low C3**, **n (%)**	28 (53,8)	0 (0)	0.024	
**Low C4**, **n(%)**	42 (80.8)	2 (33.3)	0.026	
**C3 (absolute value)**	88.3 ± 24.0	128.2 ± 18.0	< 0.001	
**C4 (absolute value)**	14.3 ± 6.2	26.9 ± 10.1	< 0.001	

c-SLE: childhood onset Systemic Lupus Erithematosus; IDF: International Diabetes Federation; SBP: systolic blood pressure; DPB: diastolic blood pressure; SLEDAI: Systemic Lupus Erythematosus Disease Activity Index; SDI: Slicc damage index. ESR: erythrocyte sedimentation rate; CRP: C-reactive protein; HDL-c: high-density lipoprotein cholesterol; LDL-c: low-density lipoprotein cholesterol

^1^Categorical data expressed as absolute counts and percentages in parentheses. Quantitative data expressed as mean ± standard deviation or as median and interquartile range in parentheses

*The chi-square test or Fisher’s exact test was used for categorical data and Student’s t-test for continuous data

**Table 4:** Sociodemographic and economic characteristics, lifestyle habits, and comorbidities of SLE patients according to the presence of metabolic syndrome as defined by ABRAN criteria

		Metabolic syndrome - ABRAN		
		**No** (*n* = 53)	**Yes** (*n* = 5)	p-value*
**Sex**				0.106
Female , n (%)		48 (90.6)	3 (60.0)	
Male , n (%)		5 (9.4)	2 (40.0)	
**Age (years)**		16.2 ± 2.6	14.7 ± 1.6	0.142
**Ethnicity**				0.615
Non-Caucasian , n (%)		44 (83)	4 (80.0)	
Caucasian , n (%)		9 (17.0)	1 (20.0)	
**Household income**				0.330
Low-income , n (%)		46 (93.9)	4 (80)	
**SBP**		108.8 ± 10.1	120.8 ± 17.9	0.099
**DBP**		71.0 ± 9.1	73.8 ± 21.7	0.462
**BMI (kg/m2)**		23.1 ± 5.0	24.5 ± 5.7	0.533
**Waist Circumference (cm)**		79.0 ± 12.4	86.1 ± 13.0	0.233
**Overweightness** n (%)		10 (18.8)	2 (40)	0.118
**Obesity** , n (%)		8 (15)	1 (20)	0.212
**Sedentary lifestyle**, **n(%)**		37 (69.8)	3 (60)	0.641
**Diagnosis time (months)**		35.0 (18.0; 52.0)	10.0 (9.0; 12.0)	0.036
**Duration of symptoms (months)**		40.0 (22.0; 72.0)	11.0 (10.0; 18.0)	0.028
**Current steroid dose (mg)**		0.0 (0.0; 20.0)	15.0 (0.0; 50.0)	0.275
**Cummulative steroid dose (mg)**		15,320 (10001; 26878)	17,520 (6370; 23560)	0.625
**SLEDAI – 2 K**		4.0 (2.0; 8.0)	8.0 (2.0; 14.0)	0.466
**SDI**		0.0 (0.0; 0.0)	0.0 (0.0; 2.0)	0.231
**ESR (mm)**		19.0 (11.0; 35.0)	10.0 (4.0; 13.0)	0.039
**CRP**		0.9 (0.5; 1.7)	1.1 (0.7; 4.5)	0.867
**Creatinine (mg/dL)**		0.6 (0.6; 0.7)	0.9 (0.6; 0.9)	0.144
**Urea (mg/dL)**		22.0 (18.0; 32.0)	38.0 (38.0; 47.0)	0.086
**Spot urine protein/creatinine ratio**		0.1 (0.1; 0.2)	0.9 (0.1; 3.1)	0.142
**Fasting blood glucose (mg/dL)**		83.2 ± 9.1	92.0 ± 18.9	0.382
**Glycated hemoglobin (%)**		5.4 ± 0.5	5.4 ± 0.5	0.934
**Insulin (microIU/mL)**		9.7 (6.8; 14.1)	11.2 (10.4; 14.8)	0.258
**Total cholesterol (mg/dL)**		152.9 ± 42.9	212.8 ± 49.8	0.013
**HDL-c (mg/dL)**	47.7 ± 14.5		72.2 ± 19.1	0.007
**LDL-c (mg/dL)**	86.4 ± 31.6		100.2 ± 38.3	0.375
**Triglycerides (mg/dL)**	95.0 ± 40.4		201.8 ± 70.7	0.001
**Albumin (g/dL)**	4.3 ± 0.4		3.6 ± 0.6	0.016
**Low C3**, **n (%)**	27 (50.9)		1 (20)	0.354
**Low C4**, **n (%)**	41 (77.4)		3 (60)	0.585
**C3 (absolute value)**	89.8 ± 25.2		120.2 ± 23.6	0.017
**C4 (absolute value)**	14.5 ± 6.4		27.0 ± 10.9	0.010

c-SLE: childhood onset Systemic Lupus Erithematosus; ABRAN: Brazilian Association of Nutrology; SBP: systolic blood pressure; DPB: diastolic blood pressure; SLEDAI: Systemic Lupus Erythematosus Disease Activity Index; SDI: Slicc damage index. ESR: erythrocyte sedimentation rate; CRP: C-reactive protein; HDL-c: high-density lipoprotein cholesterol; LDL-c: low-density lipoprotein cholesterol

Categorical data expressed as absolute counts and percentages in parentheses. Quantitative data expressed as mean ± standard deviation

*The chi-square test or Fisher’s exact test was used for categorical data and Student’s t-test for continuous data

Individuals defined as having MetS by the IDF criteria had a higher mean WC (91.1 cm ± 10.1 vs. 78.3 cm ± 12.2, *p* = 0.019), and associations between other anthropometric measures and MetS (IDF and ABRAN) were not observed. The diagnosis time and duration of symptoms were lower in patients with MetS according to ABRAN criteria 40 mo [ 22–72] vs. 11 mo [10–18], *p* = 0.036 and 35 mo [18–52] vs. 10 mo [ 9–12], *p* = 0.028, respectively).

We observed that patients with MetS had significantily higher levels of complement fractions C3 and C4 (128.2 mg/dL ± 18.0 vs. 88.3 ± 24, *p* < 0.001 and 26.9 mg/dL ± 10.1 vs. 14.3 ± 6.2, *p* < 0.001, respectively) according to the IDF criteria, and (120.2 ± 23.6 vs. 89.8 ± 25, *p* = 0,017 and 25 ± 10.9 vs. 14.5 ± 6.4, *p* = 0.010, respectively) according to the ABRAN criteria. Multivariable logistic regression analysis (Table 5) demonstrated that higher C4 levels (odds ratio (OR) = 32.6; 95% confidence interval (CI) = 1.9–544.0; *p* = 0.015) and an increased leukocyte count (per each increment of 1,000 cells/µL), OR = 1.9; 95%CI = 1.1–3.2, *p* = 0.022) were independently associated with the presence of MetS according to the IDF criteria.

**Table 5:** Logistic regression analysis of factors associated with metabolic syndrome (IDF Criteria) in cSLE patients

	Metabolic Syndrome (IDF)	
	Odds ratio (IC 95%)	*p*
**Initial model**		
Sex (male)	4.4 (0.003; 5740.7)	0.685
Age (per year)	0.6 (0.2; 1.4)	0.220
Leukocytes (per each increment of 1,000 cells/µL)	2.5 (0.9; 6.6)	0.060
Serum creatinine (mg/dL)	1.2 (0.3; 4.2)	0.797
Complement C3 (≥ 90 mg/dL)	28000048.5 (0; ∞)	> 0.999
Complement C4 (≥ 20 mg/dL)	53.5 (0.8; 3303.5)	0.058
**Final model**		
Leukocytes (per each increment of 1,000 cells/µL)	1.9 (1.1; 3.2)	**0.022**
Complement C4 (≥ 20 mg/dL)	32.6 (1.9; 544.0)	**0.015**

IDF: International Diabetes Federation, c-SLE: childhood onset Systemic Lupus Erithematosus

A backward stepwise method was used until achieve final model

Patients with MetS according to ABRAN criteria also exhibited lower ESR levels (10.0 mm [4.0–13.0] vs. 10.0 mm [11.0–35.0], *p* = 0.039), higher total cholesterol (212.8 ± 49.8 mg/dL vs. 152.9 ± 42.9 mg/dL, *p* = 0.013), HDL-c (72.2 ± 19.1 mg/dL vs. 47.7 ± 14.5 mg/dL, *p* = 0.007), and triglycerides (201.8 ± 70.7 mg/dL vs. 95 ± 40.4 mg/dL, *p* = 0.001), as well as lower albumin levels (3.6 ± 0.6 g/dL vs. 4.3 ± 0.4 g/dL, *p* = 0.016). Patients with MetS according to IDF criteria had higher SDI scores (1 [0-2.8] vs. 0 [ 0–0], *p* = 0.039).

No significant association with MetS was observed regarding cSLE clinical manifestations, current disease activity or autoantibody profile and there was no association between MetS and current or cumulative corticosteroid use in this study.

## Discussion

To our knowledge, this is the first study to assess the prevalence of MetS in cSLE patients within a predominantly low-income population, and the first to apply the new ABRAN criteria for MetS, specifically designed for use in Brazilian children and adolescents. In addition, this study also incorporates the evaluation of insulin levels and concomitant analysis of several cardiovascular risk factor and their potential relationship with laboratory variables in cSLE patients yielded meaningful results.

The prevalence of MetS in our sample was significantly lower than that reported in previous publications [7, 8], but without statistical significance with our control group. A contributing factor could be the distinct sociodemographic aspects of our cSLE and the small size of our sample. Sinicato et al. included children and adolescents from southeastern Brazil [8], while the present study involved children and adolescents from northeastern Brazil. According to the Brazilian Institute of Geography and Statistics (IBGE), the Northeast region has a low-income percentage of 38.2%, whereas the Southeast region has a percentage of 13.5% [16]. Population studies suggest that socioeconomic factors may influence the occurrence of MetS in such way that children from a higher social class are at a higher risk [17–20], contradicting what is observed in adults [21–23]. Importantly, the prevalence of MetS is higher in adults compared to cSLE. Nonetheless, traditional cardiovascular risk factors alone do not explain the high prevalence of MetS in these adults, who demonstrably have accelerated atherosclerosis and increased arterial stiffness, with a cardiovascular risk 7 to 17 times higher than the general population [24–26].

In our sample, frequence of sedentary lifestyle was similar among patients with cSLE and control subjects and the same occurred between cSLE individuals with and without MetS. Despite been a known modifiable factor with a positive impact on cardiovascular risk, sedentary lifestyle did not appear to be an independent predictor for the development of metabolic syndrome in our analysis. The role of sedentary lifestyle as a risk factor in SLE patients is debated and a prospective study that evaluated coronary artery disease (CAD) outcomes and predictive factors in patients with SLE and matched healthy controls identified that it was a predictive factor for CAD only in the control group, but not in the SLE group. This suggests that, although sedentary behavior is a general risk factor for CAD, SLE-specific factors may play a more significant role in the development of CAD in these patients [43].

Additionally, the low concordance in the diagnosis of MetS between the different criteria observed in this study illustrates the difficulty in standardizing the classification of this condition in pediatric patients, so far, there is still no universal consensus on which criteria should be applied [4, 14, 16–17]. The prevalence of MetS in Brazilian children and adolescents, according to the IDF definition, has been found to range from 1.7 to 7.9%, depending on the region [18, 19, 27, 28, 29]. Compared to this data, the prevalence of MetS observed in our cSLE patients was higher (10.3% by IDF criteria and 9% by ABRAN criteria). Sinicato et al. found a 17.1% prevalence of MetS [8] and. Gomez-Marcial et al. found a 32% prevalence of MetS in Mexican cSLE patients [7]. Notably, there are still no population or specific studies published using ABRAN criteria.

Importantly, the careful selection of patients included in this study provided a better analysis of the influence of cSLE on the prevalence of MetS, as overlapping autoimmune rheumatological diseases could influence this outcome, as studies in adults have demonstrated a high prevalence of MetS in patients with other rheumatic diseases [26, 30, 31]. In children, studies are scarce; however, they show that dyslipidemia, overweightness, and obesity are prevalent in patients with juvenile idiopathic arthritis, not solely attributed to the use of corticosteroids [32–36]. A study demonstrated that the prevalence of MetS in children and adolescents with psoriatic arthritis was of 47.17% in a sample of 460 patients and observed a significantly higher prevalence of MetS in the group of patients who had never used anti-TNF [37].

Regarding the different definitions adopted, our analysis showed that subjects with MetS, as defined by ABRAN criteria, exhibited more associations with altered lipid profiles and a shorter duration of symptoms and diagnosis time. In contrast, patients with MetS defined according to IDF criteria showed higher associations with elevated WC and SDI. These results could be partially attributed to criteria bias, as the ABRAN definition utilizes lower cut-off values for dyslipidemia and a larger WC is mandatory for the definition of MetS according to IDF.

In contrast with what has been reported in the literature [38], we observed elevated HDL-c levels in our patients with MetS. This discrepancy may be attributed to the fact that these patients also exhibited higher total cholesterol values, including triglycerides, characteristic of MetS. A previous study on MetS in cSLE patients did not show significant differences in HDL-c values between patients with and without MetS [8].

Surprisingly, we did not observe an association between lupus nephritis and MetS, as demonstrated in previous studies [8, 22], or with current disease activity. However, we did observe an association with higher SDI, which had not yet been demonstrated in research with cSLE but has already been reported in several studies conducted in adult patients with SLE [22, 23, 39, 40]. In addition, Sanchez-Perez et al. found that elevated SDI over time contributes to RI independently [41], reinforcing that MetS may accompany irreversible damage in SLE, amplifying the chronic inflammatory process and increasing the thrombotic process mediated by plasminogen activator inhibitor-1 (PAI-1) [39, 40].

This is the first study to report elevated complement fractions levels in cSLE patients with MetS. Supporting this association, a previous study also reported increased C3 levels in adult patients with SLE and MetS [42], while another study demonstrated elevated levels of complement components along with other inflammatory biomarkers, such as homocysteine, IL-8, and sICAM-1, in SLE patients with MetS [25]. Our findings are also justified by the pathophysiology of MetS, wherein the excess of visceral adiposity leads to increased production of adipocytokines, such as leptin, promoting a chronic subclinical proinflammatory state. Moreover, our multivariate analysis demonstrated that elevated leukocyte count is an independent predictor of MetS, reflecting a state of low-grade subclinical inflammation mediated by the release of TNF-α, IL-6, and C3 within adipose tissue. This chronic inflammatory environment drives sustained activation of the innate immune system, characterized by increase in leukocyte counts, particularly neutrophils and monocytes [26].

Our study has some limitations: (i) the small overall sample size and the limited number of MetS cases may have reduced the statistical power of the study to detect significant associations; and (ii) we were unable to match the household income of our control group to our patients group, although, recognizing the impact that social status has on childhood MetS, it would probably not change our outcome. We acknowledge that a larger sample would have made drawing conclusions regarding the association between MetS and clinical variables in cSLE patients more feasible. Therefore, the results of the logistic regression analysis should be interpreted with caution and validated in larger cohorts to confirm these preliminary findings.

Finally, the prevalence of Mets in the patients with cSLE seems to be low. There was association of MetS with higher cumulative damage indices and higher levels of complement. We did not observe any association with clinical manifestations, autoantibody profile and dose of corticosteroids. Nevertheless, more studies, ideally including a larger sample, are warranted to explore more specific aspects of the relationship between SLE and MetS in the pediatric population.

## Data Availability

The data that support the finding of this study are available on a reasonable request from the corresponding author.

## Abbreviations

SLE: Systemic lupus erythematosus
MetS: Metabolic syndrome
cSLE: Childhood onset systemic lupus erithematosus
IR: Insulin resistance
IDF: International Diabetes Federation
ABRAN: The Brazilian Association of Nutrology
DM: Diabetes mellitus
SAH: Systemic arterial hypertension
SBP: Systolic blood pressure
DBP: Diastolic blood pressure
BMI: Body mass index
WC: Waist circumference
SLEDAI: Systemic Lupus Erythematosus Activity Index 2000
SDI: Slicc damage index score
TG: Triglyceride
HDL-c: High-density lipoprotein cholesterol
LDL: Low-density lipoprotein cholesterol
ESR: Erythrocyte sedimentation rate
CRP: C-reactive protein
BP: Blood pressure
WC: Waist circumference
PAI-1: Plasminogen activator inhibitor-1
